# Cardiovascular disease and lung cancer

**DOI:** 10.3389/fonc.2024.1258991

**Published:** 2024-02-12

**Authors:** Mikhail de Jesus, Anindita Chanda, Titas Grabauskas, Manish Kumar, Agnes S. Kim

**Affiliations:** ^1^ Department of Cardiology, University of Connecticut Hartford Hospital, Hartford, CT, United States; ^2^ Department of Internal Medicine, University of Connecticut School of Medicine, Farmington, CT, United States; ^3^ University of Connecticut School of Medicine, Farmington, CT, United States; ^4^ Department of Cardiology, Pat & Jim Calhoun Cardiology Center, University of Connecticut School of Medicine, Farmington, CT, United States

**Keywords:** cardiac adverse events, cardiotoxicities, lung cancer, non-small cell lung cancer, small cell lung cancer, chemotherapy, immunotherapy

## Abstract

Lung cancer is the second most common cancer worldwide and the leading cause of cancer-related death. While survival rates have improved with advancements in cancer therapeutics, additional health challenges have surfaced. Cardiovascular disease (CVD) is a leading cause of morbidity and mortality in patients with lung cancer. CVD and lung cancer share many risk factors, such as smoking, hypertension, diabetes, advanced age, and obesity. Optimal management of this patient population requires a full understanding of the potential cardiovascular (CV) complications of lung cancer treatment. This review outlines the common shared risk factors, the spectrum of cardiotoxicities associated with lung cancer therapeutics, and prevention and management of short- and long-term CVD in patients with non-small cell (NSCLC) and small cell (SCLC) lung cancer. Due to the medical complexity of these patients, multidisciplinary collaborative care among oncologists, cardiologists, primary care physicians, and other providers is essential.

## Introduction

Lung cancer is the second most common malignancy and the leading cause of cancer death in the U.S and worldwide (1, 2). The American Cancer Society estimates 238,340 new diagnoses and 127,000 lung cancer-related deaths in 2023 (2). Although these numbers highlight the significant disease burden of lung cancer worldwide, there are reasons for optimism. From 1999-2019, the age-adjusted annual rate of new lung cancer decreased from 70.8 to 52.9 per 100,000 people, and the age-adjusted annual rate of death decreased from 55.4 to 33.4 per 100,000 people (3). This downturn in the rate of new cancers is likely due to the decline in smoking prevalence (1). Moreover, due to advancements in chemotherapeutics, the 5-year relative survival rate has increased from 11.7% in 1975 to 22.9% in 2018 (4).

As patients live longer due to advancements in early cancer detection and effective anticancer therapies, other medical challenges arise. One such challenge is the development of coexistent comorbidities, paramount of which is CVD. CVD has been identified as the second leading cause of death in patients with NSCLC (5). In this paper, we review the shared risks factors for developing lung cancer and CVD and discuss in depth the therapies for lung cancer that have adverse CV effects. Furthermore, we explore strategies for prevention, management, and surveillance of CVD in patients with NSCLC and SCLC.

## Shared risk factors for lung cancer and cardiovascular disease

CVD and lung cancer share a number of risk factors: smoking, hypertension, diabetes mellitus (DM), advanced age, obesity, and racial and socioeconomic status (SES) (6–9). Preexisting CV comorbidities in lung cancer patients are common given these overlapping risk factors (10, 11). A population-based study found hypertension, arrhythmia, coronary artery disease (CAD), dyslipidemia, and heart failure (HF) to be the most prevalent CV conditions in patients with lung and bronchus cancer (10). Coexistent CVD portends worse prognosis in these patients (10). Patients with NSCLC and comorbid HF, myocardial infarction (MI), or cardiac arrhythmias had the lowest overall survival (11). Interestingly, lung cancer was found to be an independent risk factor for the development of CVD, specifically CAD and MI, in a meta-analysis (6). Additionally, low SES is a well-documented risk factor for both lung cancer and CVD (9).

There are notable health inequities observed in lung cancer and CVD across various races and SES groups (9). In the United States, non-Caucasians are at an increased risk of developing lung cancer and dying from CVD (9). African Americans have 50-70% greater risk of developing HF compared with Caucasians, and African American men are 12% more likely to develop lung cancer compared with Caucasian men (12, 13). The incidence rate for lung cancer in African American population has been estimated to be 71.2/100,000 compared with 35.1–65./100,000 in other racial groups (14). Lower SES has been associated with increased smoking, lack of exercise, and lower life expectancy (15). One pooled analysis of case-control studies found a correlation between low SES and lung cancer. The highest effect was observed in men in the lowest vs. highest SES category: calculated OR for lung cancer was 1.84, 95% CI: 1.61–2.09. For women, OR was 1.54, 95% CI: 1.20–1.98 (16). Population-based studies have yielded similar results with CVD showing that lower income levels, lower educational attainment, and unemployment increase the risk of developing CVD and lead to worse clinical outcomes (17). These studies highlight the complex interrelationship between CVD and lung cancer.

### Smoking

Smoking is the single most important risk factor for lung cancer. It is estimated that male smokers are 23 times, and female smokers 13 times, more likely than never smokers to develop lung cancer (18). Individuals exposed to second-hand smoke have a 20 to 30 percent greater risk of developing lung cancer than those without exposure (19). An estimated two-thirds of lung cancer deaths worldwide are attributable to smoking (1). Cigarette smoking and its effects on the CV system are well-studied. Smoking increases the risk of atherosclerotic disease, acute coronary syndrome, stroke, and sudden death (20). Smoking has multiple deleterious effects on the CV system including reduction of nitric oxide (NO) leading to vasomotor dysfunction, pro-thrombogenic effects, alteration of lipid metabolism (increase in oxidative LDL), increased inflammation and oxidative stress (20). Smoking dramatically increases the risk of hypertension and insulin resistance, which eventually lead to the development of CVD (21, 22). The World Health Organization have promoted effective frameworks to tackle the tobacco epidemic. In the U.S., smoking rates declined from 42.4% among adults in the year 1965 to 13.7% in 2018 (23). Despite these efforts, cigarette smoking remains the leading preventable cause of death and disability (24).

Smoking-related side effects are predominantly due to endothelial cell damage (25). Inhaled transition metals, carbon monoxide, and aldehydes among other chemicals cause vasoconstriction through a decrease in NO synthesis and production of free radicals, reactive oxygen and nitrogen species (ROS and RNS) (25). The free radicals trigger a cycle of inflammation to generate more ROS and RNS and further damage the endothelium (26). These oxidative processes lead to lipid peroxidation, contributing to the development of cholesterol plaques (26). The injured endothelium upregulates adhesion molecules and recruits immune cells, further reinforcing the inflammatory state (27). The chronic inflammatory state is a favorable environment for cancer development (28, 29).

## Adverse cardiovascular effects of lung cancer therapy

Antineoplastic regimens for both NSCLC and SCLC, including immune checkpoint inhibitors, targeted therapies, cytotoxic chemotherapy, or mediastinal radiation can have profound CV effects. Arrhythmia, left ventricular (LV) dysfunction, HF, hypertension, ischemia/MI, pulmonary hypertension, thromboembolic disease, and pericarditis have been reported as side effects associated with anticancer treatment (30). Common CV adverse effects of anticancer regimens for NSCLC and SCLC are summarized in **Table 1**.

**Table 1:** Cardiovascular adverse effects of NSCLC and SCLC treatments.

Treatment Class	Examplar Drugs	Cardiac Adverse Effects
Immune checkpoint inhibitors	Pembrolizumab, Nivolumab, Atezolizumab, Durvalumab	myocarditis, pericarditis, vasculitis, conduction delay, complete heart block, atrial fibrillation, HF, MI, elevated troponin, elevated BNP, arrhythmias
Targeted therapies		
EGFR inhibitors	Erlotinib, Gefitinib, Osimertinib*	QT prolongation, HF, SVT
BRAF inhibitor	Dabrafenib	HF
MEK inhibitor	Trametinib	HF
ALK inhibitor	Brigatinib, Crizotinib, Certinib, Alectinib	conduction disease**
VEGF inhibitor	Bevacizumab	arterial HTN, HF, atrial fibrillation, arterial thromboembolic events, Takotsubo cardiomyopathy
Cytotoxic agents		
Platinum	Cisplatin^a^	thromboembolic events, elevated cardiac enzymes, MI, HF, LV hypertrophy, atrial fibrillation
Anti-nucleoside	Gemcitabine^a^	thromboembolic events
Vinca alkaloids^a^		ST elevation, MI
Anti-folate	Pemetrexed^a^	MI, thromboembolism
Taxanes	Paclitaxel, Docetaxel	bradycardia, asymptomatic left bundle branch block, ventricular tachycardia, AV conduction delay
Anthracycline	Doxorubicin	cardiomyopathy, HF

*Out of the EGFR inhibitors, osimertinib had the most significant association with the listed adverse effects

**Conduction disease defined by Waliany et al.^17^ as bradycardia, sinus node dysfunction, AV node block, bundle branch block

a The cardiac effects of cisplatin, vinca alkaloids, and pemetrexed were often observed in conjunction with the use of another therapy modality

### Immune checkpoint inhibitors

Traditionally, platinum-based dual chemotherapy had been the first-line treatment modality for NSCLC and SCLC. Immune checkpoint inhibitors (ICIs), whether used as monotherapy or combined with other forms of anticancer therapy, are now becoming first-line treatment options for lung cancer patients who are negative for driver mutations (31).

ICIs are a class of cancer therapeutics that activate the host immune system to eliminate cancer cells. They target one of the following immune checkpoints: programmed death-1 (PD-1) and its ligand (PD-L1), cytotoxic T-lymphocyte associated protein 4 (CTLA-4), and lymphocyte-activation gene 3 (LAG3). Commonly used ICIs for lung cancer are nivolumab, pembrolizumab, ipilimumab, atezolizumab, and durvalumab (**Table 2A**) (32). As the use of ICIs has become more widespread, awareness of ICI cardiotoxicity has increased (34).

**Table 2A:** U.S. Food and Drug Administration-approved immune checkpoint inhibitors as of April 2022 for the treatment of lung cancer (32, 33).

Lung Cancer	Immune Checkpoint Inhibitor	Target	Scenario
Non-small cell lung cancer	Pembrolizumab	PD-1 inhibitor	Advanced or metastatic
	Nivolumab	PD-1 inhibitor	Advanced or metastatic Neoadjuvant
	Cemiplimab	PD-1 inhibitor	Advanced or metastatic
	Atezolizumab	PD-L1 inhibitor	Advanced or metastatic Adjuvant
	Durvalumab	PD-L1 inhibitor	Adjuvant
	Ipilimumab and Nivolumab	CTLA-4 inhibitor/PD-1 inhibitor	Advanced or metastatic
Small cell lung cancer	Atezolizumab	PD-L1 inhibitor	Advanced or metastatic
	Durvalumab	PD-L1 inhibitor	Advanced or metastatic
Pleural Mesothelioma	Ipilimumab and Nivolumab	CTLA-4 inhibitor/PD-1 inhibitor	Advanced or metastatic

CV adverse events associated with ICIs include myocarditis, pericarditis, arrhythmias, heart failure, MI, ischemic stroke, venous thromboembolism, and dyslipidemia (**Table 2B**) (32, 34, 35). Chitturi and colleagues conducted a retrospective analysis to determine if ICIs are associated with an increased risk of major adverse cardiac events (MACE) in a cohort of 252 patients with lung cancer (37). They did not find a statistically significant difference in MACE incidence between the ICI cohort and non-ICI cohort (HR: 1.18, 95% confidence interval [CI]: 0.57 to 2.43; p = 0.66). However, they did find that the predominant MACE in the ICI cohort were CV death, fatal MI, and cardiac arrest. Additionally, they noted that patients receiving ICI were more likely to have an elevation of brain natriuretic peptide (BNP) and troponin I (TnI) (34). Interestingly, a recent matched cohort study revealed that ICI treatment was associated with a 3-fold increase in the risk of atherosclerotic CV events, including MI, coronary revascularization, and ischemic stroke. In addition, there was >3-fold increase in the rate of aortic atherosclerotic plaque volume after ICI therapy (38). A recent systematic review and meta-analysis of 48 randomized clinical trials showed that CV adverse effects, such as dyslipidemia, ischemic stroke, heart failure, and MI, were more common after ICI use than myocarditis (35).

**Table 2B:** Estimated incidence of cardiovascular adverse events associated with immune checkpoint inhibitor therapy based on a safety meta-analysis (35, 36).

Cardiovascular Adverse Event	Summary Incidence (95% CI)
Myocarditis	0.3% (0.2 – 0.5%)
Pericarditis	0.8% (0.6 – 1.1%)
Arrhythmias	1%
Heart Failure	0.9% (0.7 – 1.1%)
Myocardial Infarction	0.7% (0.6 – 0.9%)
Ischemic Stroke	0.9% (0.7% – 1.1%)
Venous Thromboembolism	12.9%
Dyslipidemia	1.9% (0.7 – 5.4%)

A retrospective cohort study by Jain et al. utilized a large longitudinal real-world database to assess the incidence and clinical characteristics associated with CV adverse effects (CVAEs) in patients with any cancer treated with ICI vs non-ICI (39). Within the ICI cohort, lung cancer (43.1%) was the most common cancer type. They found that anti-CTLA-4 used as monotherapy or in combination with anti-PD-1 increased the risk of HF: combination therapy (HR: 2.0, 95% CI: 1.31-3.04) and monotherapy (HR: 1.9, CI: 1.27-2.84) (39). Comorbidities including hypertension, history of MI, DM, and peripheral vascular disease also increased the risk of HF. Johnson et al. conducted a large safety database review and found a highly significant increase in the incidence of myocarditis in combination nivolumab and ipilimumab therapy versus nivolumab monotherapy (0.27% vs. 0.06%; p<0.001; 5 fatal events vs. 1) (40).

### EGFR inhibitors

Epidermal growth factor receptor (EGFR) tyrosine kinase inhibitors (TKI) are used for the treatment of advanced EGFR-mutated NSCLC. Commonly used EGFR TKIs include osimertinib, erlotinib, gefitinib, and afatinib (30). Osimertinib increases the risk of QT prolongation, HF, and atrial fibrillation when compared to other EGFR TKIs such as erlotinib, afatinib, and gefitinib (41). A comprehensive meta-analysis by Waliany et al. found that osimertinib is associated with supraventricular tachycardia in addition to QT prolongation and HF (42). Ewer et al. performed an *ad-hoc* and pooled analyses of data from clinical trials including FLAURA and AURA3 trials investigating the risk of cardiac failure in patients receiving osimertinib (43). They found a decrease in LV ejection fraction of greater than 10% to an absolute percentage point of < 50% in 3.1% and 5.5% of patients, respectively. These events were noted to be asymptomatic and resolved without treatment or need for discontinuing osimertinib (43).

Gefitinib has been reported to have an increased odds of conduction disease (ROR: 2.17, 99% CI: 1.14–4.14) compared with other EGFR inhibitors (42). One study found that pancreatic cancer patients receiving treatment with a combination of erlotinib and gemcitabine had an increase in the incidence of MI and ischemia compared with gemcitabine alone (30, 44). Analyses investigating the risk of HF due to afatinib did not find an increased risk (45).

### BRAF and MEK inhibitors

NSCLC patients with BRAF positive mutations can be treated with BRAF inhibitors. Patients who have resistant mechanisms against BRAF inhibitors are treated concomitantly with MEK inhibitors (46). BRAF inhibitors used to treat melanoma and colorectal cancer have been found to prolong the QT interval (46). Dabrafenib (BRAF inhibitor) and trametinib (MEK inhibitor) are used in combination to treat NSCLC. This combination has been associated with an increased odds of HF and arterial hypertension compared with monotherapy and other targeted therapies for NSCLC (42, 47).

### ALK inhibitors

Roughly 5% of patients with NSCLC have anaplastic lymphoma kinase (ALK) gene mutations (48, 49). ALK inhibitors (e.g., brigatinib, crizotinib, ceritinib, alectinib) have been in use since 2011 to target NSCLC with ALK mutations (48). Ehrenstein et al. performed a safety cohort study for patients receiving crizotinib (n= 456) versus the TKI erlotinib (n=2957) in Europe (EU) and USA (summarized in **Table 3**). The USA cohort treated with crizotinib had a greater cumulative incidence of prolonged QT interval-related events, bradycardia, and cardiac failure compared with EU. However, there was a significant difference in baseline characteristics between the EU and USA populations. The prevalence of comorbidities was higher in the USA group (49). Waliany et al. investigated QT prolongation resulting from targeted therapies for NSCLC (summarized in **Table 4**) (42).

**Table 3:** Two-year cumulative incidence of cardiac adverse events in patients with primary NSCLC cancer treated with crizotinib (49).

Cardiac Adverse Event	Population	Cumulative incidence rates (95% CI), %
Bradycardia	EU	1.1 (0.0-3.0)
	USA	16.9 (10.5-24.7)
QT interval prolongation related events	EU	1.0 (0.0-3.1)
	USA	26.8 (17.6 - 36.9)
Cardiac failure	EU	0.5 (0.0 – 2.1)
	USA	6.8 (3.4 – 11.7)

**Table 4:** The effects of targeted NSCLC therapies on QT interval (42).

Targeted Therapy	Odds of QT Prolongation	99% CI
ALK inhibitors vs. EGFR/BRAF inhibitors combined	ROR 5.16	3.92 – 6.81
Crizotinib vs. other ALK inhibitors	ROR 1.91	1.22 – 3.00
Ceritinib vs. other targeted NSCLC therapies	ROR 3.43	2.02 – 5.81

### VEGF inhibitors

Arterial hypertension is a well-known side-effect of vascular endothelial growth factor (VEGF) inhibitors; it was first observed in the clinical trials for bevacizumab (50). A subsequent study of bevacizumab found a dose-dependent risk of developing hypertension from bevacizumab (51). Meta-analyses have noted arterial hypertension as an adverse effect of other VEGFI as well (52, 53). In addition to dose-dependence, studies have found that hypertension occurs rapidly after drug initiation and reverses quickly upon discontinuation (54).

Aside from hypertension, congestive heart failure (CHF) is another potential risk of treatment with bevacizumab (55). Choueiri et al. completed a meta-analysis of randomized trials with bevacizumab in patients with breast cancer. They found that patients treated with bevacizumab (n = 2366) had an overall CHF incidence of 1.6% (95% CI:1.0-2.6%) compared to an overall incidence of 0.4% (95% CI:0.2-1.0%) in the chemotherapy group without bevacizumab. The relative risk of CHF in the bevacizumab group was 4.74 (95% CI:1.16-11.18; p=0.001). Additionally, there have been two reported cases of Takotsubo cardiomyopathy believed to have been caused by bevacizumab (56). Both cases involved male patients receiving bevacizumab, one for colon cancer and the other for metastatic NSCLC (56).

Bevacizumab is also associated with an increased risk for arterial thromboembolic events. In a *post hoc* analysis of RCTs studying bevacizumab in patients with cancer, including NSCLC, Scappaticci et al. found that bevacizumab and chemotherapy combination was associated with an increased risk for arterial thromboembolic events compared to chemotherapy alone (HR: 2.0, 95% CI: 1.05-3.75, p = 0.031) (57).

### Cytotoxic agents

Cisplatin is associated with an increased risk of acute coronary syndrome (30). This risk is higher in older patients (over 65) and in those receiving concomitant radiotherapy (30, 58). In addition, cisplatin increases the risk of both venous and arterial thromboembolic events (30). Late complications in cancer survivors treated with cisplatin include hypertension, LV diastolic dysfunction, and ischemic heart disease (30, 59). Gemcitabine has been associated with thrombotic microangiopathy and hypertension (30).

A meta-analysis of RCTs comparing vinorelbine with other chemotherapies did not find a difference in the risk of cardiac events in patients receiving vinorelbine compared with other chemotherapeutic regimens (60). Pemetrexed, an antifolate cytotoxic agent, in conjunction with platinum therapy is a first-line treatment for non-squamous NSCLC (61). Cardiotoxicity occurs primarily when pemetrexed is used with other cytotoxic medications, such as cisplatin (30). Taxanes, especially in conjunction with trastuzumab, bevacizumab, or platinum therapy, have been associated with conduction abnormalities, such as bradycardia, asymptomatic left bundle branch block, or ventricular tachycardia (30, 62, 63).

Anthracycline chemotherapy has a limited role in the treatment of metastatic SCLC, and its use has a well-established link to cardiotoxicity (64). The diagnosis of anthracycline-induced cardiotoxicity (AICT) is typically made when there is new-onset clinical HF or asymptomatic LV dysfunction (65, 66). Studies using various definitions of AICT estimate the incidence rate to be 2.2-9% (65, 66). Patients who have received high-dose anthracyclines (cumulative doxorubicin ≥250 mg/m^2^ or epirubicin ≥600 mg/m^2^) are at a high risk of AICT (65, 66). Additional risk factors for AICT include history of underlying CVD, hypertension, DM, obesity, genetic susceptibility, and concomitant exposure to another cardiotoxic drug and/or radiation (65, 66). Less common side effects of anthracycline are arrhythmia and pericarditis (67).

### Radiotherapy

In the treatment of lung cancer, radiotherapy (RT) can be used concurrently with chemotherapy, prior to or after surgical resection, or for palliative reasons (68). Due to the anatomic proximity of the heart to the lungs, cardiac tissue may be inadvertently irradiated. RT can lead to potential adverse CV toxicities, including coronary artery disease, conduction system abnormalities, valvular heart disease, pericardial disease, and non-ischemic cardiomyopathy (68, 69). Risk of developing CV toxicity after RT is closely linked to the mean heart dose (MHD), a reflection of cardiac radiation exposure, and also depends on dose distribution and exposure of specific cardiac substructures (70). Generally, >15 to 25 Gy MHD is considered high risk, and >25 Gy MHD confers very high risk (70). Additionally, underlying CV risk factors and concomitant exposure to doxorubicin impact the risk of developing radiation-induced heart disease (RIHD) (70). Baseline characteristics such as receiving radiation at a younger age, smoking, pre-existing CAD, hypertension, hyperlipidemia, and post-menopausal state are additional important risk factors (71).

The pathophysiology of RIHD has been extensively reviewed elsewhere (71–73). *Via* formation of toxic free radicals, mediastinal RT can lead to endothelial injury, inflammation, platelet aggregation, thrombosis, and atherosclerotic plaque development in coronary arteries (71, 74). Furthermore, RT can cause fibrosis, thickening, and calcification of cardiac valves, leading to regurgitation and/or stenosis (71–73). Inflammation and fibrosis can also affect the conduction system, myocardium, and pericardial tissue (71–73). Given the high prevalence of smoking, CAD, and other CV risk factors, the lung cancer population is likely more vulnerable to RIHD. More studies should be performed to better understand the prevalence, natural history, prevention, and treatment of RIHD in patients with lung cancer.

## Cardiovascular risk factor modification in patients with lung cancer

Given the high prevalence of preexisting CVD and CV risk factors in patients with lung cancer, management of these patients is particularly challenging. Patients with underlying CV conditions should have them optimally managed prior to starting cancer treatment. Smoking cessation using a combination of behavioral interventions together with pharmacotherapy should be recommended (75). Underlying hypertension, DM, and hyperlipidemia should be optimally managed prior to, during, and after cancer treatment. Cancer survivors should continue to be screened for modifiable CV risk factors as these patients are more likely to be underdiagnosed and undertreated compared with the general population (76, 77). Patients with a history of cancer are less likely to receive cardioprotective therapies, especially antiplatelets and statins (77).

Management of BP is paramount because hypertension is highly prevalent in lung cancer patients and accounts for more ASCVD deaths than any other modifiable risk factor (10, 11, 75). The 2019 ACC/AHA guidelines recommend controlling BP to <130/80 for patients with hypertension and ASCVD or 10-year ASCVD risk ≥10% (75). These recommendations for BP targets are extrapolated from the general population and not well-studied in patients with cancer. Initial management of hypertension involves a low sodium diet and a minimum of 150 minutes of moderate-intensity, or 75 minutes of vigorous-intensity, aerobic exercise per week (75). The choice of antihypertensive regimen is dependent on patient characteristics and comorbidities similar to the general population (78). Patients with known diabetes, diabetic nephropathy, proteinuria, or chronic kidney disease (CKD) should be managed with an angiotensin converting enzyme inhibitors (ACE-I) or angiotensin II receptor blockers (ARB). Patients with CHF and LV systolic dysfunction should receive guideline-directed medical therapy, i.e. ACE-I/ARB/angiotensin receptor-neprolysin inhibitor (ARNI), sodium-glucose cotransporter-2 (SGLT2) inhibitor, beta blocker (BB), and mineralocorticoid receptor antagonist (MRA) as tolerated. For those with CAD, BB, ACE-I/ARB, and/or nitrates are considered (78). Certain classes of antihypertensive medications are preferred depending on the anticancer regimen used. For example, for VEGFI-induced hypertension, ACE-I/ARB, dihydropyridine calcium channel blocker (CCB), and/or thiazide/thiazide-like diuretic are first-line agents (78, 79).

The European Society of Medical Oncology (ESMO) 2020 guidelines suggest that patients receiving cardiotoxic anticancer therapies, e.g., anthracyclines and/or trastuzumab, are considered to have stage A HF and thus should be treated with ACE-I/ARB and/or beta blockers (e.g., carvedilol or nebivolol) to protect against cardiotoxicity (80). The 2022 ACC/AHA/HFSA guidelines recommend that patients with type 2 DM and history of ASCVD or high ASCVD risk receive SGLT2 inhibitors to prevent hospitalization for HF (81). Stage B HF patients with LVEF < 40% should be managed with ACE-I/ARB and beta blocker (81).

Hyperlipidemia is another highly prevalent CV risk factor among lung cancer patients (11). Similar to hypertension, the current recommendations for lipid screening and management are extrapolated from the general population due to lack of specific data in cancer patients. The 2018 ACC/AHA guidelines recommend routinely screening patients between the ages of 40-75 and consider it reasonable to screen patients aged 20-39 every 4 to 6 years. There are currently no guidelines to direct the timing of screening in patients with a history of cancer or those actively receiving cancer treatment. Initial steps for the management of hyperlipidemia include dietary and lifestyle changes (75). Statins are the cornerstone of pharmacotherapy management for dyslipidemia. Statin initiation is currently recommended in primary prevention for patients with an LDL-C ≥190 mg/dL, patients between 40 and 75 years of age with DM, and patients between 40 and 75 years of age and 10-year ASCVD risk ≥20% (75). High-intensity statin therapy is indicated for secondary prevention of ASCVD. Non-statin therapies, such as ezetimibe or proprotein convertase subtilisin/kexin type 9 serine protease (PCSK9) inhibitors, should be started in patients with established ASCVD on maximally tolerated statin therapy with an LDL-C ≥70 mg/dL (82). There is evidence that hyperlipidemia contributes to inflammation in cancer patients. ESMO 2020 guidelines recommend that patients be continued on treatment for hyperlipidemia while receiving chemotherapy and to consider initiation of statin in patients with concomitant CAD (80).

## Cardiovascular disease screening and surveillance in patients with lung cancer

For patients with lung cancer who will receive cardiotoxic anticancer therapies, a multidisciplinary collaboration among cardiology, oncology, radiation oncology, PCP, and pharmacology is essential to minimize CV toxicity while allowing cancer treatment to proceed without interruption (65, 70, 80). For most lung cancer patients, it is recommended to perform a baseline CV risk assessment and evaluation, which includes physical examination, BP measurement, ECG, lipid panel and hemoglobin A1c (HbA1c), and smoking status assessment. Since patients with cancer undergo serial blood draws, lipid profile and HbA1c can be easily added for screening and monitoring. As discussed above, modifiable CV risk factors should be optimally controlled prior to initiation of anticancer therapy (65, 70, 80). Patients with lung cancer undergo non-gated chest CT scans for cancer staging and surveillance. Incidental detection of coronary artery calcification is prevalent and can be helpful in providing CAD risk stratification and guiding prevention strategies (83). BP measurements that are routinely performed at oncology visits should be assessed and followed for the screening and monitoring of hypertension (84). In addition, established CVD should be managed according to relevant ACC/AHA and ESC guidelines before, during, and after antineoplastic therapy (65, 70, 80).

For patients with lung cancer receiving ICI, strategies for outpatient monitoring of ICI myocarditis are not well-defined. Most cardio-oncology programs use a symptom-based approach and do not routinely check biomarkers and echocardiogram (echo) on all patients receiving ICI because of the relatively low incidence (0.04% to 1.14%) of ICI myocarditis (85, 86). Lee Chuy et al. monitored ECG and troponin levels in all patients treated with combination ICI therapy (87). Among 76 consecutive patients, no overt or subclinical myocarditis was detected (87). However, it is generally recommended to check a baseline ECG and measure biomarkers (BNP and cardiac troponin (cTn)) prior to ICI therapy initiation (70). Baseline echo can be considered in high-risk patients before starting ICI. Risk factors for developing ICI cardiotoxicity include treatment with dual ICI (e.g., anti–CTLA-4 plus anti–PD-1) or ICI in combination with another cardiotoxic agent, noncardiac immune-related adverse events, and prior history of CVD. Currently, there are international efforts to better understand the risk factors and the full clinical spectrum of ICI myocarditis (88). It is recommended to repeat CV assessment every 6-12 months in high-risk patients who require long-term ICI treatment (70).

For patients presenting with a clinical suspicion for ICI myocarditis, prompt initiation of workup, which includes ECG, troponin, BNP, CRP, echo, cardiac MRI, and consideration of endomyocardial biopsy, is important (80, 89). The presence of concomitant noncardiac immune-related adverse events, such as myositis or nephritis, in addition to cardiac symptoms raises the clinical suspicion of ICI myocarditis. While pursuing the diagnostic workup, it is generally recommended to start steroids (1000 mg IV methylprednisolone daily for 3 days) (89). In a case series of clinically suspected ICI myocarditis (n= 126), those patients who received corticosteroids within 24 hours of presentation regardless of dose showed the best outcome, while those who received corticosteroids after 72 hours showed the worst outcome (90). Additionally, high-dose corticosteroid administration was associated with a 73% lower risk of major adverse cardiac events (90). If the diagnostic workup reveals definite or probable myocarditis, steroids should be continued and tapered over at least 4-6 weeks depending on clinical and biomarker response (89).

Before starting VEGFI therapy, patients with lung cancer should be informed about the risk of developing new-onset or worsening hypertension so that they can participate in BP monitoring. BP measurement must be obtained in all patients at baseline and every subsequent clinical visit (54, 70, 78). This is necessary not only to detect an exaggerated hypertensive response with VEGFI, but also to identify patients with preexisting hypertension who could benefit from early BP management (54, 70, 78). In addition, regular home BP monitoring (HBPM) during the first cycle, after each increase of VEGFI dose, and every 2-3 weeks thereafter is recommended (70). In patients treated with VEGFI at elevated risk of QTc prolongation (e.g., vandetanib, sorafenib, and sunitinib), ECG should be performed monthly during the first 3 months and every 3-6 months thereafter (70). Baseline echo is recommended in high- and very high-risk patients treated with VEGFI to rule out underlying cardiomyopathy. Serial follow-up echos should be considered in moderate- and high-risk patients (70). The role of cardiac biomarkers in VEGFI cardiotoxicity detection is currently not well-defined.

Similar to other anticancer regimens above, lung cancer patients receiving ALK or EGFR inhibitor should undergo baseline CV risk assessment (70). Because osimertinib has the potential to cause clinical HF or asymptomatic LV dysfunction, baseline echo is recommended in all patients before starting osimertinib (70). Serial echo imaging every 3 months can be considered during therapy. For early detection of the hypertensive side effect, HBPM should be considered for patients treated with brigatinib, crizotinib, or lorlatinib (70). Since ALK inhibitor therapy is associated with QT prolongation and conduction disease, ECG should be considered 4 weeks after starting therapy and every 3-6 months during treatment (70). Guidelines for the prevention and monitoring of anthracycline induced cardiotoxicity have been extensively reviewed elsewhere in previous literature. **Table 5** summarizes the key recommendations for the surveillance of cardiotoxicity during treatment for lung cancer.

**Table 5:** Key recommendations for the screening and surveillance of cardiotoxicity during treatment for lung cancer (70).

Treatment Class	Key Recommendations
Immune checkpoint inhibitors	*All patients:* • Check baseline ECG and biomarkers (BNP, cTn). • Monitor for symptoms/signs of myocarditis. *High-risk patients: • Consider checking baseline echo. • Repeat CV assessment every 6-12 months.
VEGF inhibitors	*All patients:* • Check BP at baseline and every clinical visit. • Regular HBPM during the first cycle, after each VEGFI dose increase, and every 2-3 weeks thereafter. *For VEGFI at increased risk of QT prolongation (e.g. vandetanib, sorafenib, and sunitinib):* • Check ECG monthly during the first 3 months and every 3-6 months thereafter. **High- and very high-risk patients: • Check baseline and serial echoes.
ALK or EGFR inhibitors	*All patients before starting Osimertinib:* • Check baseline echo. • Consider serial echo every 3 months. *Patients treated with brigatinib, crizotinib, or lorlatinib:* • Consider HBPM *Patients on ALK inhibitor therapy:* • Check ECG 4 weeks after starting therapy and every 3-6 months thereafter.
Anthracycline	*All patients:* • Check baseline echo. • Repeat echo within 12 months after completing therapy. **Moderate-risk patients: • Check echo after a cumulative dose of >250 mg/m^2^ of doxorubicin or equivalent. **High- and very high-risk patients: • Check echo every 2 cycles and within 3 months of treatment completion. • Check baseline BNP and cTn. • Check BNP and cTn before every cycle and 3 and 12 months after completing therapy.

*Administration of dual ICI, combination ICI-cardiotoxic therapy, presence of noncardiac immune-related adverse events, prior cancer therapy-related cardiac dysfunction, or CVD (70).

**Risk based on Heart Failure Association-International Cardio-Oncology Society (HFA-ICOS) cardio-oncology cardiovascular risk assessment tool prior to cardiotoxic anticancer therapy (70, 91). This risk calculator includes the following data: previous history of CVD, cardiac biomarkers, age, cardiovascular risk factors, previous cardiotoxic treatment, and lifestyle risk factors.

## Conclusion

In patients with lung cancer, concomitant CV comorbidities are exceedingly common due to overlapping risk factors: smoking, hypertension, DM, advanced age, and obesity. Coexistent CVD portends poor overall and oncologic prognosis in patients with lung cancer. This review highlights the complex interrelationship between CVD and lung cancer. Furthermore, many of the anticancer regimens used for the treatment of NSCLC and SCLC can potentially cause adverse CV effects. To improve the overall survival of lung cancer patients, it is critical to understand the cardiotoxicities associated with modern treatment regimens. More data are needed on how to prevent, surveil, and treat CV adverse effects due to novel anticancer therapies. A multidisciplinary collaboration among cardiology, oncology, radiation oncology, PCP, and pharmacology is critical to minimize CV toxicity while allowing cancer treatment to proceed without interruption.

## Author contributions

MDJ: Writing – original draft, Writing – review & editing. AC: Writing – original draft, Writing – review & editing. TG: Writing – original draft, Writing – review & editing. MK: Supervision, Writing – review & editing. AK: Conceptualization, Supervision, Writing – review & editing.

## References

[1] Sung H Ferlay J Siegel RL Laversanne M Soerjomataram I Jemal A. Global cancer statistics 2020: GLOBOCAN estimates of incidence and mortality worldwide for 36 cancers in 185 countries. CA Cancer J Clin (2021) 71(3):209–49. doi: 10.3322/caac.21660 33538338

[2] Jemal A Siegel R Kratzer TB. Cancer statistics center. Am Cancer Soc. doi: 10.3322/caac.21763

[3] U.S. Cancer Statistics Working Group. U.S. Cancer Statistics Data Visualizations Tool, based on 2021 submission data (1999-2019). U.S. Department of Health and Human Services, Centers for Disease Control and Prevention and National Cancer Institute (2022). Available at: https://www.cdc.gov/cancer/dataviz.

[4] Surveillance, Epidemiology, and End Results (SEER) Program. SEER*Stat Database: Age-Adjusted SEER Incidence and U.S. Death Rates and 5-Year Relative Survival (Percent) By Primary Cancer Site, Sex and Time Period, National Cancer Institute, DCCPS, Surveillance Research Program.

[5] Sun JY Zhang ZY Qu Q Wang N Zhang YM Miao LF. Cardiovascular disease-specific mortality in 270,618 patients with non-small cell lung cancer. Int J Cardiol (2021) 330:186–93. doi: 10.1016/j.ijcard.2021.02.025 33581175

[6] Yuan M Li QG. Lung cancer and risk of cardiovascular disease: A meta-analysis of cohort studies. J Cardiothorac Vasc Anesth (2018) 32(1):e25–7. doi: 10.1053/j.jvca.2017.04.033 29150236

[7] Handy CE Quispe R Pinto X Blaha MJ Blumenthal RS Michos ED. Synergistic opportunities in the interplay between cancer screening and cardiovascular disease risk assessment: together we are stronger. Circulation (2018) 138(7):727–34. doi: 10.1161/CIRCULATIONAHA.118.035516 30359131

[8] Koene R Prizment A Blaes A Konety S. Shared risk factors in cardiovascular disease and cancer. Circulation (2016) 133(11):1104–14. doi: 10.1161/CIRCULATIONAHA.115.020406 PMC4800750 26976915

[9] State of Lung Cancer | Racial and Ethnic Disparities. American Lung Association (2022). Available at: https://www.lung.org/research/state-of-lung-cancer/racial-and-ethnic-disparities.

[10] Liu D Ma Z Yang J Zhao M Ao H Zheng X. Prevalence and prognosis significance of cardiovascular disease in cancer patients: a population-based study. Aging (2019) 11(18):7948–60. doi: 10.18632/aging.102301 PMC6781987 31562288

[11] Kravchenko J Berry M Arbeev K Lyerly HK Yashin A Akushevich I. Cardiovascular comorbidities and survival of lung cancer patients: Medicare data based analysis. Lung Cancer (2015) 88(1):85–93. doi: 10.1016/j.lungcan.2015.01.006 25704956 PMC4375130

[12] Cleveland Clinic. In: Heart Disease Risk: How Race and Ethnicity Play a Role. Available at: https://my.clevelandclinic.org/health/articles/23051-ethnicity-and-heart-disease.

[13] Lung Cancer Statistics | How Common is Lung Cancer? (2022). Available at: https://www.cancer.org/cancer/lung-cancer/about/key-statistics.html.

[14] Zavala VA Bracci PM Carethers JM Carvajal-Carmona L Coggins NB Cruz-Correa MR. Cancer health disparities in racial/ethnic minorities in the United States. Br J Cancer (2021) 124(2):315–32. doi: 10.1038/s41416-020-01038-6 PMC7852513 32901135

[15] NATA. Socioeconomic Status and Health Care (2018). Available at: https://www.nata.org/blog/Jordan-grantham/socioeconomic-status-and-its-impact-health-care.

[16] Hovanec J Siemiatycki J Conway DI Olsson A Stücker I Guida F. Lung cancer and socioeconomic status in a pooled analysis of case-control studies. PloS One (2018) 13(2):e0192999. doi: 10.1371/journal.pone.0192999 29462211 PMC5819792

[17] Schultz WM Kelli HM Lisko JC Varghese T Shen J Sandesara P. Socioeconomic Status and cardiovascular outcomes: challenges and interventions. Circulation (2018) 137(20):2166–78. doi: 10.1161/CIRCULATIONAHA.117.029652 PMC5958918 29760227

[18] U.S. Department of Health and Human Services. The Health Consequences of Smoking: A Report of the Surgeon General. (Centers for Disease Control and Prevention) (2004).

[19] U.S. Department Of Health And Human Services. The Health Consequences of Involuntary Exposure to Tobacco Smoke A Report of the Surgeon General. (Centers for Disease Control and Prevention) (2006).

[20] Ambrose JA Barua RJ. The pathophysiology of cigarette smoking and cardiovascular disease: an update. J Am Coll Cardiol (2004) 43(10):1731–7. doi: 10.1016/j.jacc.2003.12.047 15145091

[21] Skipina TM Soliman EZ Upadhya B. Association between secondhand smoke exposure and hypertension: nearly as large as smoking. J Hypertens (2020) 38(10):1899–908. doi: 10.1097/HJH.0000000000002478 32890262

[22] Bergman BC Perreault L Hunerdosse D Kerege A Playdon M Samek AM. Novel and reversible mechanisms of smoking-induced insulin resistance in humans. Diabetes (2012) 61(12):3156–66. doi: 10.2337/db12-0418 PMC3501865 22966072

[23] Centers for Disease Control and Prevention. National Center for Health Statistics. National Health Interview Survey 1965-2018. Analysis for years 1997-2018 by the American Lung Association Research Team using SPSS software. (U.S. Department of Health and Human Services) (2023).

[24] U.S. Department of Health and Human Services. The Health Consequences of Smoking—50 Years of Progress: A Report of the Surgeon General. U.S. Department of Health and Human Services, Centers for Disease Control and Prevention, National Center for Chronic Disease Prevention and Health Promotion, Office on Smoking and Health (2014).

[25] Münzel T Hahad O Kuntic M Keaney JF Deanfield JE Daiber A. Effects of tobacco cigarettes, e-cigarettes, and waterpipe smoking on endothelial function and clinical outcomes. Eur Heart J (2020) 41(41):4057–70. doi: 10.1093/eurheartj/ehaa460 PMC7454514 32585699

[26] Caliri AW Tommasi S Besaratinia A. Relationships among smoking, oxidative stress, inflammation, macromolecular damage, and cancer. Mutat Res Rev Mutat Res (2021) 787:108365. doi: 10.1016/j.mrrev.2021.108365 34083039 PMC8287787

[27] Tesfamariam B DeFelice AF. Endothelial injury in the initiation and progression of vascular disorders. Vascul Pharmacol (2007) 46(4):229–37. doi: 10.1016/j.vph.2006.11.005 17218160

[28] Coussens LM Werb Z. Inflammation and cancer. Nature (2002) 420(6917):860–7. doi: 10.1038/nature01322 PMC2803035 12490959

[29] Risk Factors: Chronic Inflammation - NCI (2015). Available at: https://www.cancer.gov/about-cancer/causes-prevention/risk/chronic-inflammation.

[30] Zaborowska-Szmit M Krzakowski M Kowalski D Szmit S. Cardiovascular complications of systemic therapy in non-small-cell lung cancer. J Clin Med (2020) 9(5):1268. doi: 10.3390/jcm9051268 32349387 PMC7287714

[31] Choi H Lee JK Oh HJ Kim MS Kho BG Park CK. Efficacy and dose of afatinib in patients with non-small cell lung cancer after failure of prior gefitinib or erlotinib treatment. Thorac Cancer (2021) 12(10):1598–604. doi: 10.1111/1759-7714.13957 PMC8107028 33811467

[32] Tan S Day D Nicholls SJ Segelov E. Immune checkpoint inhibitor therapy in oncology: current uses and future directions: JACC: cardioOncology state-of-the-art review. JACC CardioOncol (2022) 4(5):579–97. doi: 10.1016/j.jaccao.2022.09.004 PMC9830229 36636451

[33] U.S Food and Drug Administration. Drugs@FDA: FDA-Approved Drugs. Drug Databases. U.S. Department of Health and Human Services (2022).

[34] Wang DY Okoye GD Neilan TG Johnson DB Moslehi JJ. Cardiovascular toxicities associated with cancer immunotherapies. Curr Cardiol Rep (2017) 19(3):21. doi: 10.1007/s11886-017-0835-0 28220466 PMC10176498

[35] Dolladille C Akroun J Morice PM Dompmartin A Ezine E Sassier M. Cardiovascular immunotoxicities associated with immune checkpoint inhibitors: a safety meta-analysis. Eur Heart J (2021) 42(48):4964–77. doi: 10.1093/eurheartj/ehab618 34529770

[36] Tan M Wai D Ling Chng C Hwang W. Acarbose is an effective treatment for severe hypertriglyceridemia secondary to l-asparaginase and dexamethasone. Leuk Lymphoma (2012) 53(6):1245–6. doi: 10.1016/j.jaccao.2019.11.013 22149169

[37] Chitturi KR Xu J Araujo-Gutierrez R Bhimaraj A Guha A Hussain I. Immune checkpoint inhibitor-related adverse cardiovascular events in patients with lung cancer. JACC CardioOncol (2019) 1(2):182–92. doi: 10.1016/j.jaccao.2019.11.013 PMC8352266 34396181

[38] Drobni Z Alvi R Taron J Zafar A Murphy S Rambarat P. Association between immune checkpoint inhibitors with cardiovascular events and atherosclerotic plaque. Circulation (2020) 142(24):2299–311. doi: 10.1161/CIRCULATIONAHA.120.049981 PMC7736526 33003973

[39] Jain P Bugarin JG Guha A Jain C Patil N Shen T. Cardiovascular adverse events are associated with usage of immune checkpoint inhibitors in real-world clinical data across the United States. ESMO Open (2021) 6(5):100252. doi: 10.1056/NEJMoa1609214 34461483 PMC8403739

[40] Johnson DB Balko JM Compton ML Chalkias S Gorham J Xu Y. Fulminant myocarditis with combination immune checkpoint blockade. N Engl J Med (2016) 375(18):1749–55. doi: 10.1056/NEJMoa1713137 PMC5247797 27806233

[41] Soria JC Ohe Y Vansteenkiste J Reungwetwattana T Chewaskulyong B Lee KH. Osimertinib in untreated EGFR-mutated advanced non-small-cell lung cancer. N Engl J Med (2018) 378(2):113–25. doi: 10.1016/j.jtho.2021.07.030 29151359

[42] Waliany S Zhu H Wakelee H Chen T Witteles R Neal J. Pharmacovigilance analysis of cardiac toxicities associated with targeted therapies for metastatic NSCLC. J Thorac Oncol (2021) 16(12):2029–39. doi: 10.1200/JCO.20.01171 34418561

[43] Ewer MS Tekumalla SH Walding A Atuah KN. Cardiac safety of osimertinib: A review of data. J Clin Oncol (2021) 39(4):328–37. doi: 10.1186/s40959-015-0006-7 PMC8078322 33356419

[44] Senderowicz A Johnson J Sridhara R Zimmerman P Justice R Pazdur R. Erlotinib/gemcitabine for first-line treatment of locally advanced or metastatic adenocarcinoma of the pancreas. Oncology (2007) 21(14):696–1706. doi: 10.1186/s40959-015-0006-7 18247017

[45] Ewer MS Patel K O’Brien D Lorence RM. Cardiac safety of afatinib: a review of data from clinical trials. Cardiooncology (2015) 1(1):3. doi: 10.18632/oncotarget.5853 33530147 PMC7837143

[46] Bronte E Bronte G Novo G Bronte F Bavetta MG Re GL. What links BRAF to the heart function? new insights from the cardiotoxicity of BRAF inhibitors in cancer treatment. Oncotarget (2015) 6(34):35589–601. doi: 10.1002/cam4.3938 PMC4742127 26431495

[47] Guha A Jain P Fradley MG Lenihan D Gutierrez JM Jain C. Cardiovascular adverse events associated with BRAF versus BRAF/MEK inhibitor: Cross-sectional and longitudinal analysis using two large national registries. Cancer Med (2021) 10(12):3862–72. doi: 10.1371/journal.pone.0229179 PMC8209554 33982883

[48] Elliott J Bai Z Hsieh SC Kelly SE Chen L Skidmore B. ALK inhibitors for non-small cell lung cancer: A systematic review and network meta-analysis. PloS One (2020) 15(2):e0229179. doi: 10.1002/pds.5193 32074131 PMC7029857

[49] Ehrenstein V Huang K Kahlert J Bahmanyar S Karlsson P Loflin L. Outcomes in patients with lung cancer treated with crizotinib and erlotinib in routine clinical practice: A post-authorization safety cohort study conducted in Europe and in the United States. Pharmacoepidemiol Drug Saf (2021) 30(6):758–69. doi: 10.1200/JCO.2003.10.066 33428292

[50] Kabbinavar F Hurwitz H Fehrenbacher L Meropol NJ Novotny WF Lieberman G. randomized trial comparing bevacizumab plus fluorouracil (FU)/leucovorin (LV) with FU/LV alone in patients with metastatic colorectal cancer. J Clin Oncol (2003) 21(1):60–5. doi: 10.1053/j.ajkd.2006.11.039 12506171

[51] Zhu X Wu S Dahut WL Parikh CR. Risks of proteinuria and hypertension with bevacizumab, an antibody against vascular endothelial growth factor: systematic review and meta-analysis. Am J Kidney Dis (2007) 49(2):186–93. doi: 10.1016/S1470-2045(08)70003-2 17261421

[52] Wu S Chen J Kudelka A Lu J Zhu X. Incidence and risk of hypertension with sorafenib in patients with cancer: a systematic review and meta-analysis. Lancet Oncol (2008) 9(2):117–23. doi: 10.1080/02841860802314720 18221915

[53] Zhu X Stergiopoulos K Wu S. Risk of hypertension and renal dysfunction with an angiogenesis inhibitor sunitinib: systematic review and meta-analysis. Acta Oncol (2009) 48(1):9–17. doi: 10.1161/CIRCULATIONAHA.110.992230 18752081

[54] Nazer B Humphreys BD Moslehi J. Effects of novel angiogenesis inhibitors for the treatment of cancer on the cardiovascular system: focus on hypertension. Circulation (2011) 124(15):1687–91. doi: 10.1200/JCO.2010.31.9129 21986775

[55] Choueiri TK Mayer EL Je Y Rosenberg JE Nguyen PL Azzi GR. Congestive heart failure risk in patients with breast cancer treated with bevacizumab. J Clin Oncol (2011) 29(6):632–8. doi: 10.2147/TCRM.S3960 21205755

[56] Franco TH Khan A Joshi V Thomas B. Takotsubo cardiomyopathy in two men receiving bevacizumab for metastatic cancer. Ther Clin Risk Manage (2008) 4(6):1367–70. doi: 10.1093/jnci/djm086 PMC2643117 19337443

[57] Scappaticci FA Skillings JR Holden SN Gerber HP Miller K Kabbinavar F. Arterial thromboembolic events in patients with metastatic carcinoma treated with chemotherapy and bevacizumab. J Natl Cancer Inst (2007) 99(16):1232–9. doi: 10.1053/j.seminoncol.2013.09.010 17686822

[58] Steingart RM Yadav N Manrique C Carver JR Liu J. Cancer survivorship: cardiotoxic therapy in the adult cancer patient; cardiac outcomes with recommendations for patient management. Semin Oncol (2013) 40(6):690–708. doi: 10.1200/JCO.2000.18.8.1725 24331191

[59] Meinardi M Gietema J van der Graaf W van Veldhuisen J Runne M Sluiter W. Cardiovascular morbidity in long-term survivors of metastatic testicular cancer. J Clin Oncol (2000) 18(8):1725–32. doi: 10.1046/j.0767-3981.2003.00215.x 10764433

[60] Lapeyre-Mestre M Gregoire N Bugat R Montastruc JL. Vinorelbine-related cardiac events: a meta-analysis of randomized clinical trials. Fundam Clin Pharmacol (2004) 18(1):97–105. doi: 10.1097/JTO.0b013e3182307efe 14748761

[61] Spigel DR Hainsworth JD Shipley DL Ervin TJ Kohler PC Lubiner ET. A randomized phase II trial of pemetrexed/gemcitabine/bevacizumab or pemetrexed/carboplatin/bevacizumab in the first-line treatment of elderly patients with advanced non-small cell lung cancer. J Thorac Oncol (2012) 7(1):196–202. doi: 10.1097/CAD.0000000000000080 21900836

[62] Joshi M Liu X Belani CP. Taxanes, past, present, and future impact on non-small cell lung cancer. Anticancer Drugs (2014) 25(5):571–83. doi: 10.1161/01.CIR.0000133187.74800.B9 24463482

[63] Yeh ETH Tong AT Lenihan DJ Yusuf SW Swafford J Champion C. Cardiovascular complications of cancer therapy: diagnosis, pathogenesis, and management. Circulation (2004) 109(25):3122–31. doi: 10.1016/j.jacc.2014.06.1167 15226229

[64] Vejpongsa P Yeh E. Prevention of anthracycline-induced cardiotoxicity: challenges and opportunities. J Am Coll Cardiol (2014) 64(9):938–45. doi: 10.1200/JCO.2016.70.5400 25169180

[65] Armenian S Lacchetti C Barac A Carver J Constine LS Denduluri N. Prevention and monitoring of cardiac dysfunction in survivors of adult cancers: american society of clinical oncology clinical practice guideline. J Clin Oncol (2017) 35(8):893–911. doi: 10.1136/heartjnl-2017-312103 27918725

[66] Henriksen P. Anthracycline cardiotoxicity: an update on mechanisms, monitoring and prevention. Heart (2018) 104(12):917–77. doi: 10.7326/0003-4819-125-1-199607010-00008 29217634

[67] Shan K Lincoff AM Young B. Anthracycline-induced cardiotoxicity. Ann Intern Med (1996) 125(1):47–58. doi: 10.1016/j.jacc.2019.03.500 8644988

[68] Atkins KM Rawal B Chaunzwa TL Lamba N Bitterman DS Williams CL. Cardiac radiation dose, cardiac disease, and mortality in patients with lung cancer. J Am Coll Cardiol (2019) 73(23):2976–87. doi: 10.1097/CRD.0b013e3182431c23 31196455

[69] Filopei J Frishman W. Radiation-induced heart disease. Cardiol Rev (2012) 20(4):184–8. doi: 10.1093/eurheartj/ehac244 22314140

[70] Lyon A Lopez-Fernandez T Couch L Asteggiano R Aznar M Bergler-Klein J. 2022 ESC Guidelines on cardio-oncology developed in collaboration with the European Hematology Association (EHA), the European Society for Therapeutic Radiology and Oncology (ESTRO) and the International Cardio-Oncology Society (IC-OS): Developed by the task force on cardio-oncology of the European Society of Cardiology (ESC). Eur Heart J (2022) 43(41):4229–361. doi: 10.1161/JAHA.121.021686 36017568

[71] Belzile-Dugas E Eisenberg MJ. Radiation-induced cardiovascular disease: review of an underrecognized pathology. J Am Heart Assoc (2021) 10(18):e021686. doi: 10.1007/s11936-013-0259-0 34482706 PMC8649542

[72] Mousavi N Nohria A. Radiation-induced cardiovascular disease. Curr Treat Options Cardiovasc Med (2013) 15(5):507–17. doi: 10.1016/j.jacc.2017.09.1095 24092234

[73] Chang HM Okwuosa TM Scarabelli T Moudgil R Yeh ETH. Cardiovascular complications of cancer therapy: best practices in diagnosis, prevention, and management: part 2. J Am Coll Cardiol (2017) 70(20):2552–65. doi: 10.1016/j.jacbts.2018.01.014 PMC5825188 29145955

[74] Venkatesulu BP Mahadevan LS Aliru ML Yang X Bodd MH Singh PK. Radiation-induced endothelial vascular injury: A review of possible mechanisms. JACC Basic Transl Sci (2018) 3(4):563–72. doi: 10.1016/j.jacc.2019.03.010 PMC6115704 30175280

[75] Arnett DK Blumenthal RS Albert MA Buroker AB Goldberger ZD Hahn EJ. 2019 ACC/AHA guideline on the primary prevention of cardiovascular disease: A report of the american college of cardiology/american heart association task force on clinical practice guidelines. J Am Coll Cardiol (2019) 74(10):e177–232. doi: 10.1161/JAHA.121.024735 PMC7685565 30894318

[76] Chow EJ Chen Y Armstrong GT Baldwin LM Cai CR Gibson TM. Underdiagnosis and undertreatment of modifiable cardiovascular risk factors among survivors of childhood cancer. J Am Heart Assoc (2022) 11(12):e024735. doi: 10.1016/j.jaccao.2020.05.010 35674343 PMC9238650

[77] Untaru R Chen D Kelly C May A Collins NJ Leitch J. Suboptimal use of cardioprotective medications in patients with a history of cancer. JACC CardioOncol (2020) 2(2):312–5. doi: 10.1093/ckj/sfad195 PMC8352161 34396237

[78] Pandey S Kalaria A Jhaveri K Herrmann S Kim A. Management of hypertension in patients with cancer: challenges and considerations. Clin Kidney J (2023) (2):171–90. doi: 10.1093/ckj/sfad195 PMC10689173 38046043

[79] Mohammed T Singh M Tiu JG Kim AS. Etiology and management of hypertension in patients with cancer. Cardio-Oncol Lond Engl (2021) 7(1):14. doi: 10.1186/s40959-021-00101-2 PMC8022405 33823943

[80] Curigliano C Lenihan D Fradley M Ganatra S Barac A Blaes A. Management of cardiac disease in cancer patients throughout oncological treatment: ESMO consensus recommendations. Ann Oncol (2020) 31(2):171–90. doi: 10.1016/j.annonc.2019.10.023 PMC8019325 31959335

[81] Heidenreich PA Bozkurt B Aguilar D Allen LA Byun JJ Colvin MM. 2022 AHA/ACC/HFSA guideline for the management of heart failure: A report of the american college of cardiology/american heart association joint committee on clinical practice guidelines. 2022 AHAACCHFSA guidel manag heart fail rep am coll cardiol heart assoc jt comm clin pract guidel. Circulation (2022) 145(18):e895–1032. doi: 10.1016/j.jacc.2018.11.002 35363499

[82] Grundy SM Stone NJ Bailey AL Beam C Birtcher KK Blumenthal RS. 2018 AHA/ACC/AACVPR/AAPA/ABC/ACPM/ADA/AGS/APhA/ASPC/NLA/PCNA guideline on the management of blood cholesterol: A report of the american college of cardiology/american heart association task force on clinical practice guidelines. J Am Coll Cardiol (2019) 73(24):3168–209. doi: 10.1093/eurjpc/zwac185 30423391

[83] Hooks M Sandhu G Maganti T Chen KHA Wang M Cullen R. Incidental coronary calcium in cancer patients treated with anthracycline and/or trastuzumab. Eur J Prev Cardiol (2022) 29(17):2200–10. doi: 10.1038/s41371-023-00831-z 36017793

[84] Tan S Spear E Sane N Nelson AJ Nerlekar N Segelov E. Blood pressure surveillance in cancer patients treated with immune checkpoint inhibitors. J Hum Hypertens (2023) 37(11):1043–6. doi: 10.1634/theoncologist.2018-0130 PMC10632129 37076569

[85] Ganatra S Neilan T. Immune checkpoint inhibitor-associated myocarditis. Oncologist (2018) 23(8):879–86. doi: 10.1016/j.jacc.2018.02.037 PMC6156176 29802219

[86] Mahmood S Fradley M Cohen J Nohria A Reynolds K Heinzerling L. Myocarditis in patients treated with immune checkpoint inhibitors. J Am Coll Cardiol (2018) 71(16):1755–64. doi: 10.1634/theoncologist.2019-0040 PMC6196725 29567210

[87] Chuy K Oikonomou E Postow M Callahan M Chapman P Shoushtari A. Myocarditis surveillance in patients with advanced melanoma on combination immune checkpoint inhibitor therapy: the memorial sloan kettering cancer center experience. Oncologist (2019) 24(5):e196–197. doi: 10.1136/jitc-2022-004699 PMC6516118 30910868

[88] Nguyen L Bretagne M Arrondeau J Zahr N Ederhy S Abbar B. Reversal of immune-checkpoint inhibitor fulminant myocarditis using personalized-dose-adjusted abatacept and ruxolitinib: proof of concept. J Immunother Cancer (2022) 10(4):e004699. doi: 10.1016/j.jaccao.2022.11.005 35383117 PMC8984056

[89] Moslehi J Salem J. Immune checkpoint inhibitor myocarditis treatment strategies and future directions. JACC CardioOncol (2022) 4(5):704–7. doi: 10.1161/CIRCULATIONAHA.119.044703 PMC9830216 36636442

[90] Zhang L Zlotoff D Awadalla M Mahmood S Nohria A Hassan M. Major adverse cardiovascular events and the timing and dose of corticosteroids in immune checkpoint inhibitor-associated myocarditis. Circulation (2020) 141(24):2031–4. doi: 10.1002/ejhf.1920 PMC7301778 32539614

[91] Lyon AR Dent S Stanway S Earl H Brezden-Masley C Cohen-Solal A. Baseline cardiovascular risk assessment in cancer patients scheduled to receive cardiotoxic cancer therapies: a position statement and new risk assessment tools from the Cardio-Oncology Study Group of the Heart Failure Association of the European Society of Cardiology in collaboration with the International Cardio-Oncology Society. Eur J Heart Fail (2020) 22(11):1945–60. doi: 10.3109/10428194.2011.647312 PMC8019326 32463967

